# Artificial intelligence-based framework for early detection of heart disease using enhanced multilayer perceptron

**DOI:** 10.3389/frai.2024.1539588

**Published:** 2025-01-10

**Authors:** Monir Abdullah

**Affiliations:** Department of Computer Science and Artificial Intelligence, College of Computing and Information Technology, University of Bisha, Bisha, Saudi Arabia

**Keywords:** heart disease, cardiac disease, machine learning, cardiovascular diseases, multilayer perceptron, detection

## Abstract

Cardiac disease refers to diseases that affect the heart such as coronary artery diseases, arrhythmia and heart defects and is amongst the most difficult health conditions known to humanity. According to the WHO, heart disease is the foremost cause of mortality worldwide, causing an estimated 17.8 million deaths every year it consumes a significant amount of time as well as effort to figure out what is causing this, especially for medical specialists and doctors. Manual methods for detecting cardiac disease are biased and subject to medical specialist variance. In this aspect, machine learning algorithms have proved to be effective and dependable alternatives for detecting and classifying patients who are affected by heart disease. Precise and prompt detection of human heart disease can assist in avoiding heart failure within the initial stages and enhance patient survival. This study proposed a novel Enhanced Multilayer Perceptron (EMLP) framework complemented by data refinement techniques to enhance predictive accuracy. The classification model asses using the CDC cardiac disease dataset and achieved 92% accuracy by surpassing all the traditional methods. The proposed framework demonstrates significant potential for the early detection and prediction of cardiac-related diseases. Experimental results indicate that the Enhanced Multilayer Perceptron (EMLP) model outperformed the other algorithms in terms of accuracy, precision, F1-score, and recall, underscoring its efficacy in cardiac disease detection.

## 1 Introduction

Cardiac illness is one of the furthermost prevalent illnesses nowadays (Kaur and Kaur, 2023). For many healthcare professionals, the diagnosis of cardiac illness at an initial phase is very crucial to protect their patients from severe conditions and save lives. Early disease identification might save countless lives, and the death rate could be lowered if patients consume their medicines during the right period. Cardiac illnesses cover a wide range of dangers including Arrhythmia, stroke, valve disease, heart failure, and so on. These types of illnesses are becoming more widespread in younger age groups as a result of a lack of physical activity caused by lifestyle changes. Smoking, lack of physical activity, bad mental health, high cholesterol foods, unhealthy foods, and lifestyle choices are the main reasons for getting affected by heart disease. Recent advancements in machine learning and artificial intelligence (AI) have significantly improved the detection and classification of medical conditions, particularly in cardiovascular diseases. Several studies have explored the integration of AI and IoT for healthcare applications, especially for heart disease detection (Alghamedy et al., 2022). Additionally, AI-based multimodal approaches in medical imaging, such as those proposed by Bilal et al. (2024a), have shown promise for improving diagnostic accuracy.

Heart failure is a major disorder that may be the reason behind several dangerous situations in the modern world. Nearly 26 million people worldwide suffer from this type of disease each year. This disorder is a clinical illness with many etiologies in which the cardiac system cannot supply enough blood to the body's crucial organs. Doctors could use electronic medical records to detect heart failure based on the patient's symptoms and clinical laboratory tests. However, a good diagnosis of HF necessitates access to specialists and medical resources (Al-Yarimi et al., 2021), which is not always possible, making the diagnosis difficult. To save time and effort, it is essential to predict the patients' state using machine learning algorithms. We need to develop a system that would be prepared to quickly identify the symptoms of a heart stroke and prevent it, as there is a rapid increase in cardiac disease ratios in young people. Since it is unaffordable for the normal person to often conduct expensive tests such as the electrocardiogram (ECG) which is a mechanism that can accurately predict the possibility of emerging cardiac disease must be in place.

Artificial intelligence is becoming progressively common in the healthcare sector (Baashar et al., 2022), as computer examination may reduce manual faults and improve accuracy. Algorithms make illness detection extremely reliable. These techniques are utilized to forecast illnesses such as cardiac illness (Mahesh et al., 2022), liver disease (Almazroi, 2022), skin cancer, breast cancer (Sonawane and Patil, 2022), tumors, etc. For the accurate classification and prediction of heart disease conditions with limited variables, a comparison examination of several models was conducted for the detection of the cardiac disease dataset in this article. The dataset contains 18 variables including the class attribute collected from the annual CDC (Centers for Disease Control) survey dataset of 300,000 adults. K-Nearest Neighbor (KNN) (Tasnim and Habiba, 2021), Naïve Bayes (NB), Random Forest (RF), Support Vector Machine (SVM) (Dharmendra, 2022), multilayer perceptron (MLP) (Kishor and Jeberson, 2021), Decision Tree (DT) (Dharmendra, 2022), as well as Enhanced Multilayer Perceptron (EMLP) classifiers, were used in the algorithms to determine how fine the selected classification algorithms perform in classifying or detecting cardiac disease conditions.

The Enhanced Multilayer Perceptron (EMLP) model is particularly well-suited for this study due to its ability to address the limitations of traditional MLP and other models. Unlike standard MLPs, the EMLP integrates advanced techniques such as optimized weight initialization, adaptive learning rates, and additional layers tailored for feature extraction. These enhancements enable the EMLP to effectively capture complex, non-linear relationships in the data, which is critical for predicting cardiac disease conditions with high accuracy. Moreover, the EMLP's robustness to overfitting, achieved through regularization techniques such as dropout and L2 regularization, makes it highly effective in scenarios with diverse or imbalanced datasets like the one used in this study.

This study aims to predict or detect cardiac diseases by employing machine learning techniques in the optimal way possible to figure out the best-performing model on the annual CDC (Centers for Disease Control and Prevention) survey dataset among the proposed models or classifiers. This study evaluated various machine learning models along with the Enhanced Multilayer Perceptron (EMLP) on the CDC cardiac disease dataset. 92% was the highest accuracy achieved by the Enhanced Multilayer Perceptron (EMLP) model.

## 2 Related work

To gain a clearer insight into the progress made in the prediction of heart disease, the current body of literature can be classified into three principal categories: traditional methods, ensemble methods, and deep learning approaches. This classification offers a systematic overview of the various methodologies, emphasizing their development and individual contributions.

### 2.1 Traditional methods

Various approaches have been addressed by the research public for early diagnosis of heart-related diseases such as Heart Arrhythmias, Heart Attacks, and Heart Failure. Traditional machine learning techniques have been widely applied to predict and analyze cardiac illnesses. For example, David and Antony Belcy (2018) introduced a trio of prediction models consisting of a decision tree classifier, a naïve Bayes classification model, and a random forest algorithm. Among these, the random forest classifier outperformed both the decision tree and naïve Bayes classifiers in terms of accuracy. Similarly, Hashi and Zaman (2020) utilized five machine learning models for prediction and assessment based on accuracy, precision, and recall. The study highlighted the effectiveness of a logistic model tree, boosted by ADA and bagging models, with Random Forest showing the highest predictionprecision.

Other studies also leveraged traditional methods. Otoom et al. (2015) employed Naïve Bayes (NB), support vector machine (SVM), and functional trees, achieving a maximum accuracy of 84.5%. Using the same dataset, Vembandasamyp et al. (2015) focused solely on the NB classification algorithm, achieving a moderately better accuracy of 86.4%. Moreover, Usha and Kanchana (2022) explored seven machine learning classifiers, including logistic regression (LR), decision trees (DT), and random forests (RF), using a Kaggle dataset of 4240 patient records. The LR model emerged as the best performer, achieving 85.84% accuracy.

### 2.2 Ensemble methods

Ensemble techniques have been a key focus for improving cardiac disease prediction. Latha and Jeeva (2019) proposed a majority voting system involving multiple machine learning classifiers such as Naïve Bayes, multilayer perceptron (MLP), and random forest. This ensemble method showed promising results in diagnosing cardiac illnesses at early stages. Additionally, Kavitha et al. (2021) proposed a hybrid method combining decision tree and random forest models. This hybrid approach leveraged the probabilistic capabilities of random forests, resulting in improved prediction accuracy.

Another ensemble-based study (Haq et al., 2018) employed attribute selection techniques such as Relief, LASSO, and mRMR, alongside algorithms like logistic regression, KNN, and ANN. The logistic regression classifier achieved the highest prediction accuracy of 89%.

### 2.3 Deep learning approaches

Deep learning methods have significantly advanced the diagnosis of cardiac illnesses. Many existing works have focused on machine learning algorithms for disease detection, especially in medical imaging. For instance, Shafiq et al. (2023) introduced DeepSVDNet, a deep learning-based approach for diabetic retinopathy detection, which showcases the potential of deep learning frameworks in medical applications. Furthermore, Bilal et al. (2024b) presented a dual-stream feature transfer framework for ophthalmic image classification, demonstrating the effectiveness of multimodal frameworks in disease detection. Verma and Mathur (2020) developed a system utilizing deep learning to predict heart disease by selecting only relevant features, leading to improved accuracy and precision. Convolutional Neural Networks (CNNs) were also used by Raju et al. (2022), where optimized weights, activation functions, and hidden neurons enhanced the prediction rate and reduced errors.

Hybrid deep learning techniques have also been explored. For instance, Paragliola and Coronato (2020) integrated an LSTM model with a CNN network to analyze ECG signals, achieving significant improvements in detecting heart disease risk for individuals with hypertension. Furthermore, Ali et al. (2019) combined a linear SVM model with an L2-regularized variant to achieve a 3.3% performance improvement compared to traditional SVM models.

In an attempt to enhance the organization of the related work section, a summary table (Table 1) which gives a comparative analysis of previous methodologies with their drawbacks has been added. This table provide the information about which datasets was used, which models were trained and what was the maximum achieved accuracy in each paper. This brief representation reveals the usage of numerous methods that are common in heart disease detection: Decision Trees (Figure 3); Random Forest (Figure 4); Convolutional Neural Networks (Figure 5); Long-Short Term Memory (Figure 6). Also, the limitations column raises awareness of serious issues that apply to these methods including data bias, data overfitting and scant validation across various populations. And this table clearly best illustrates the need to establish a comprehensive and generalizable model which can overcome these lacks. The proposed methodology intends to fill these gaps with the help of advanced techniques and range of datasets promising a higher accuracy level.

**Table 1:** Comparison of related work.

**References**	**Dataset**	**Model(s)**	**Top accuracy (%)**	**Limitations**
David and Antony Belcy (2018)	-	DT, NB, RF	-	Lack of comprehensive dataset; limited evaluation metrics.
Latha and Jeeva (2019)	Cleveland heart dataset	NB, MLP, RF	84.49	Limited generalizability due to small sample size.
Hashi and Zaman (2020)	Heart disease UCI	LR, KNN, SVM, RF, DT	91.8	Potential overfitting due to high model complexity.
Haq et al. (2018)	Heart disease UCI	LR, KNN, ANN, SVM, NB, DT, RF	89	Results depend heavily on the choice of model parameters.
Usha and Kanchana (2022)	Cardiovascular disease dataset (Ulianova, 2019)	KNN, DT, RF, LR, SVM, NB, XGBoost	85.84	Dataset lacks diversity; potential bias in feature selection.
Otoom et al. (2015)	Kaggle (Otoom et al., 2015)	NB, SVM, Functional Trees	84.5	Limited interpretability of complex models.
Vembandasamyp et al. (2015)	Kaggle (Otoom et al., 2015)	NB	86.4	Simplistic model may not capture complex patterns.
Jindal et al. (2022)	-	LR, RF, KNN	87.5	Lack of validation on external datasets.
Louridi et al. (2019)	Heart disease UCI	SVM, KNN, NB	86.8	Potential bias from dataset used; limited metrics reported.
Ali et al. (2019)	UCI	SVM	92.22	Results may not generalize to other populations.
Verma and Mathur (2020)	-	DNN	-	High computational cost and complexity in model training.
Raju et al. (2022)	-	DNN, RNN, LSTM, CNN, CCNN, GSO-CCNN	94.99	High model complexity leads to longer training times; risk of overfitting.
Paragliola and Coronato (2020)	ECG signals (Paragliola and Coronato, 2020)	LSTM, CNN	-	Limited dataset size may affect model reliability.
Kavitha et al. (2021)	UCI	DT, RF, Hybrid (DT+RF)	88	Lack of comparative analysis with more recent models.

## 3 Material and methods

### 3.1 Methodology

**Figure 1:**
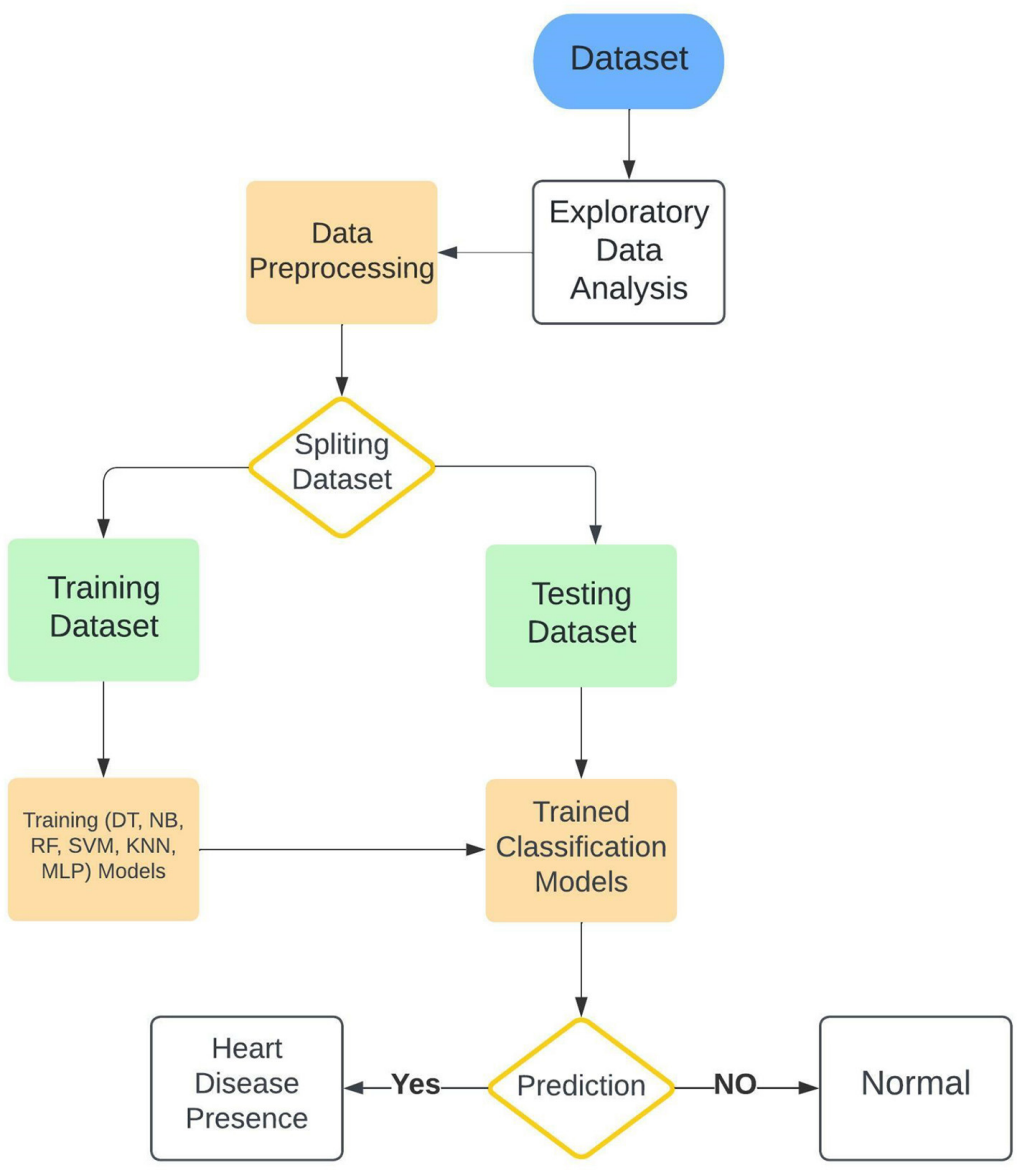
Architecture of the proposed cardiac disease prediction system that demonstrates a structured approach for early cardiac disease detection.

The current study presented several ML classification algorithms in order to assess as well as achieve the best possible combination regarding the diagnosis of cardiac diseases in relationships of performance. At the stage of preprocessing and cleaning the data, certain features were selected and optimized in order to manage to fit each model specifically. Categorical features were preprocessed and converted to features or attributes in numerical shape, furthermore, the attributes that would have been negatively contributing to the training process were removed from the training datasets in order to avoid deviating the training procedure out of the planned method. The research workflow is explained in Figure 1. In our study, we adopted a machine learning approach that builds upon the principles outlined by Tahir et al. (2022), who employed feature optimization techniques to improve model performance in physical activity recognition (Hamza et al., 2019). These methodologies are also applicable in optimizing disease detection models, such as the one presented in this work. To assess the efficiency of the presented system along with other ML algorithms different machine learning techniques were employed in this research along with the Enhanced Multilayer Perceptron (EMLP) which is a modified and improved version of the Multilayer Perceptron (MLP). All these models were trained on the same dataset to evaluate and examine the efficiency of every single classifier. Furthermore, the architecture and theoretical details of the models are explained in the classification algorithms subsection.

The workflow Refined data is processed by the Enhanced Multilayer Perceptron (EMLP) model, which analyzes complex patterns to deliver precise predictions. This framework supports early diagnosis, aiming to reduce cardiac mortality by enabling prompt medical intervention.

### 3.2 Dataset

**Figure 2:**
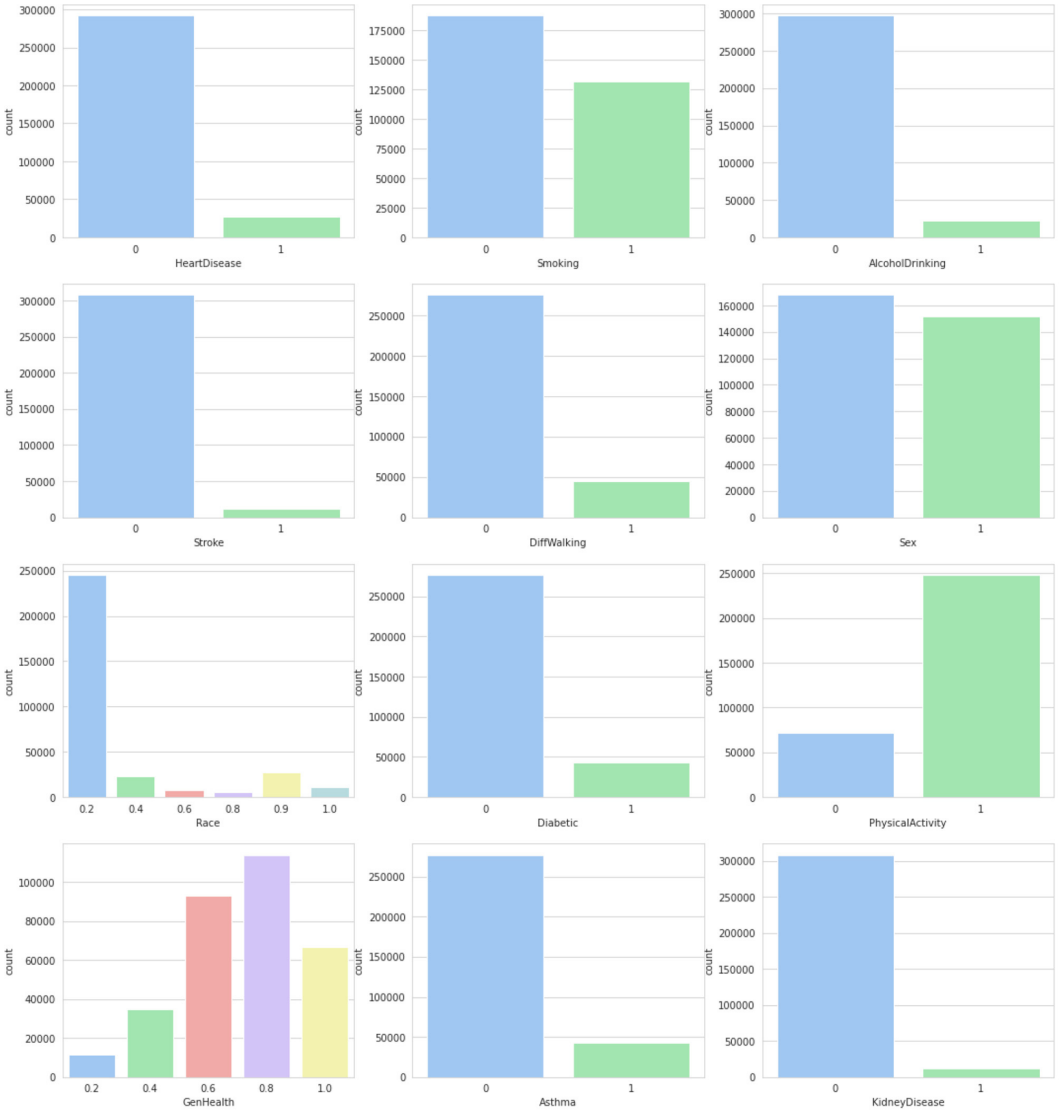
Statistical description of categorical attributes.

**Figure 3:**
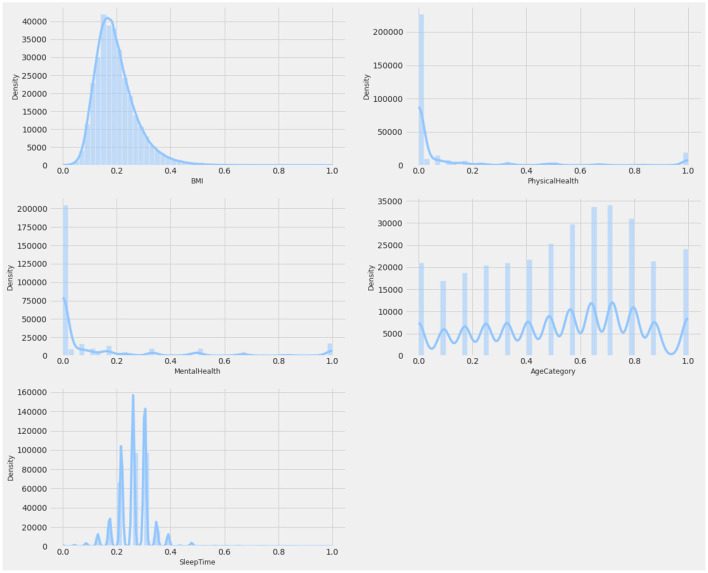
Statistical description of numerical attributes.

The CDC dataset was utilized to conduct several experiments. Details of the data are described in Table 2. This dataset comprises data from more than 300,000 adults, providing detailed information about their health status. It contains 17 attributes, including 12 categorical features and 5 numerical features. Missing values, where present, were handled using an imputation strategy based on the type of feature: mean imputation for numerical features and mode imputation for categorical features. This ensures the completeness of the data while maintaining its integrity. Figures 2, 3 provide a statistical description of the CDC (Centers for Disease Control and Prevention) dataset.

**Table 2:** Details of the CDC dataset.

**Feature**	**Value**	**Feature**	**Value**
Number of features	17	Numeric features	7
Number of patients	319,795	Missing values	-
Binary features	10	Added values	-

Attribute BMI explains the body mass index of an individual in numerical form. The Smoking feature shows whether a person has smoked a total of 100 cigarettes in his/her entire life, as it is a boolean feature having only a yes or no option. Alcohol Drinking Attribute explains whether a mature male drinks more than 14 intoxicating beverages per week or a grown female drinks more than seven intoxicating beverages per week. The stroke feature indicates if a person suffered or had a stroke in his/her entire life for at least one time.

Physical Health attribute shows how many days in the past 30 days a person has suffered from illness or had any type of injuries. The Mental Health feature explains from the last 30 days, how many days a person had a bad mental health status or his/her mental health wasn't good. The diffWaking feature explains whether a person has any type of difficulty in walking or climbing stairs.

The Gender feature indicates a person's sex whether it's male or female. Age Category attribute indicates the age ranging from 18 years up to above 80. Race attribute contains information regarding the ethnicity of an individual containing five race categories White, Black, Asian, American Indian/Alaskan Native, or Other. The diabetic feature shows if an individual is suffering from diabetes. The Physical Activity attribute is a boolean feature that indicates whether an individual is doing any type of health activity such as running, walking, or working out or not. The GenHealth attribute contains information about the overall condition of the health of an individual ranging from poor, fair, good, very good, to excellent. The sleep Time feature contains information about the average sleep time in hours of an individual ranging from 5 to 23 h per day. Asthma is a two-option attribute that explains if an individual is suffering from asthma illness or not. Kidney Disease is a binary feature that has a value of 1 if an individual is suffering from any kind of kidney disease otherwise the value would be 0. The Skin Cancer feature exposes whether a person is suffering from skin cancer or not as it has two options (Yes, No). The dataset description is mentioned in Table 3.

**Table 3:** Dataset description.

**Attribute**	**Description**
BMI	Body Mass Index, ranging from 12 to 94.8
Smoking	Yes or No
Alcohol drinking	Yes or No
Stroke	Yes or No
Physical health	Ranging from 0 to 30
Mental health	Ranging from 0 to 30
Difficulty in walking	Yes or No
Gender	Male or Female
Age category	Ranging from 18 to above 80
Race	“White”, “Black”, “Asian”, “American Indian/Alaskan Native”, or “Other”
Diabetic	Yes or No
Physical activity	Yes or No
General health	“Very Good”, “Good”, “Fair”, “Poor”, or “Excellent”
Sleep time	Ranging from 1 to 23 hours
Asthma	Yes or No
Kidney disease	Yes or No
Skin cancer	Yes or No

**Figure 4:**
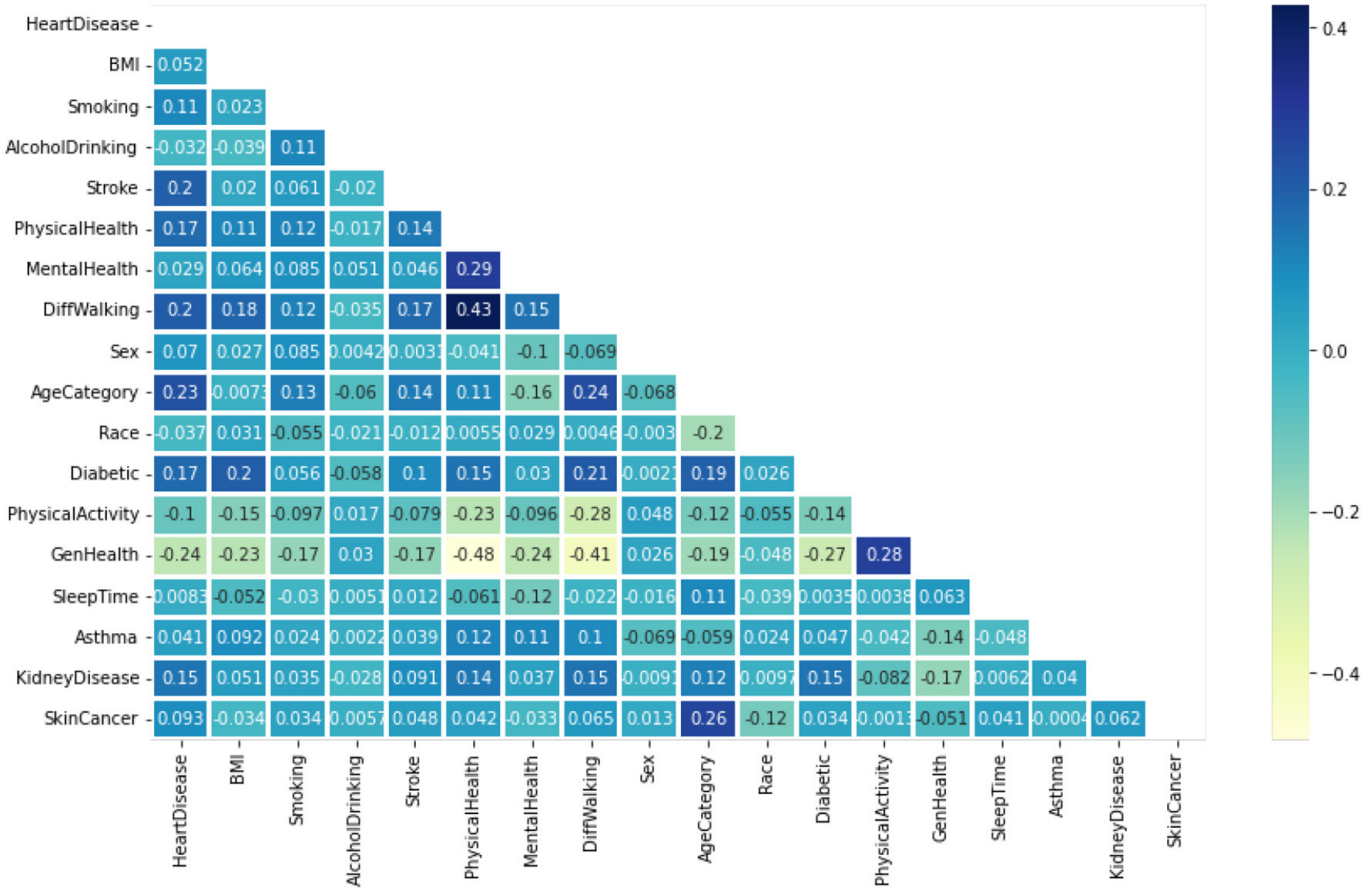
Features heatmap of the dataset.

Figure 4 displays the heatmaps. These are graphic demonstrations of correlation matrices that demonstrate links between numerous variables (Almazroi, 2022). The relationship coefficient might include any quantity between -1 and 1. A correlation is the type of statistical word expressing an association between a couple of variables that are linearly correlated. It's known as the correlation measure of a couple of parameters. The purpose of this situation is to discover a correlation between numerous factors and afterwards consolidate the outcomes.

### 3.3 Preprocessing

Most of the attributes had to be processed to make the dataset ready for training and evaluating the classification algorithms. The Age Category feature in the dataset in its raw form was categorical and had to be converted to a numerical feature to be processed and thought to the classification algorithms. On the other hand, when speaking about the multilayer perceptron model all the categorical features that contained “Yes” and “No” options had to be converted to 1 or 0 respectively, likewise in the Gender category, the “Male” and “Female” options had to be converted into numerical values in the form of 1 and 0 to be fed to MLP and the EMLP models.

For the attributes that were in numerical form but ranging more than 0 and 1, a scaling function was applied to that type of attribute that would shrink down the huge numerical values to values ranging from 0 to 1 to be easily processed by the classification algorithms. Eventually, all the attributes were scaled to have numerical values ranging from 0 to 1 depending upon the attribute type.

### 3.4 Classification algorithms

All the classification models that have been trained and utilized for the experiments are demonstrated and explained in this section.

#### 3.4.1 Decision tree (DT)

**Figure 5:**
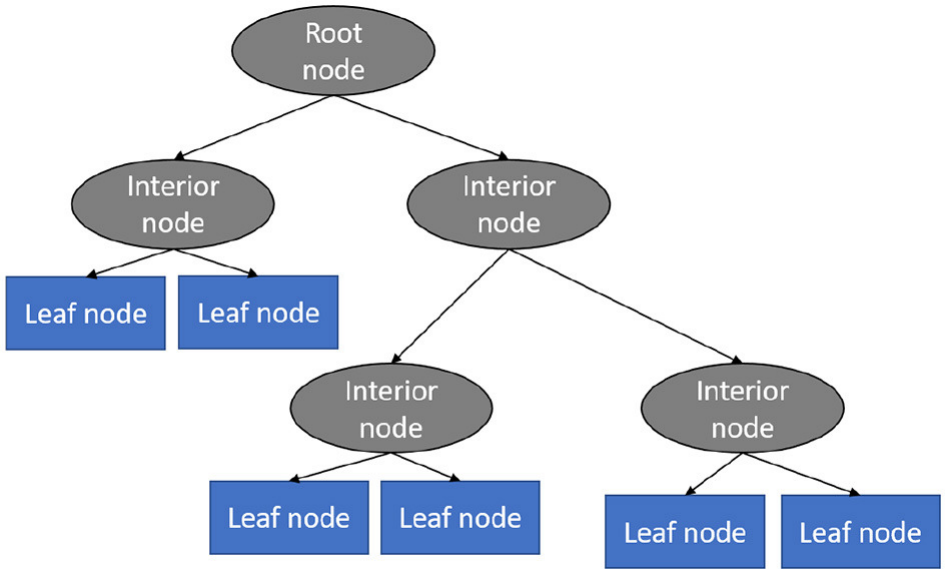
Decision tree algorithm (Vidhya, 2023b).

This technique is a supervised learning technique and it is one of the best as well as most often used models for categorization and prediction (Charbuty and Abdulazeez, 2021). In a decision tree, which looks like a flowchart, every interior node specifies an assessment of a feature, each division demonstrates the examination's outcome, and every leaf node includes a category tag. Within a decision tree, there exists a couple of nodes, the choice node, and the leaf node. Choice nodes are employed to make decisions and include many divisions, where leaf nodes represent the results of the choices (Kumar, 2022). Figure 5 shows the different components of the explained algorithm.

This classification model predicts cases by categorizing them along the chart from the root to some leaf node that offers the case's prediction or classification (Riyaz et al., 2022). Beginning at the origin node of the chart, a case is predicted by examining the feature indicated by the node, afterwards going along the chart division according to the value of the feature. This method is then repeated for the new node's subtree.

#### 3.4.2 Naïve Bayes (NB)

This classification model is a supervised classification algorithm that works based on the Bayes formula. It is a probabilistic classification model that classifies or performs predictions based on the probability of an entity (Marathe et al., 2021). The Naive Bayes algorithm computes the posterior probability of the feature or the category by utilizing the equation shown in Equation 1.


(1) $$\begin{array}{l} P\left(c|I\right)=\frac{P\left(I|c\right)P\left(c\right)}{P\left(I\right)} \end{array}$$

It can perform prediction efficiently such as disease prediction, spam filtering, and classification of documents. Naive Bayes is a frequently used model as it helps build rapid machine learning models that can perform rapid predictions.

#### 3.4.3 K-nearest neighbor (KNN)

This is a simple classification algorithm that employs the supervised learning method. It is included among the most frequently utilized models as it is easy to implement and utilize (Yunus et al., 2021). It is a non-parametric classification model, which means it does not create any expectations regarding the fundamental entities.

K-Nearest Neighbor (KNN) categorizes the entities based on common similarity within the data points. K-Nearest Neighbor Computes the Euclidean distance in-between K count of neighboring points and considers the K nearest neighbors based on Euclidean space calculated by Equation 2. It is called a lazy learner algorithm as well since it does not rapidly acquire any patterns from the training chunk, as an alternative, it saves the training entities afterwards it takes an action based on it throughout the classification process. Through the training stage, the KNN classification model saves the data and afterwards categorizes it into a class that is similar to the new entities.


(2) $$\begin{array}{l} d=\sqrt{\left(X2-X1\right)+\left(Y1-Y2\right)} \end{array}$$

#### 3.4.4 Random forest (RF)

**Figure 6:**
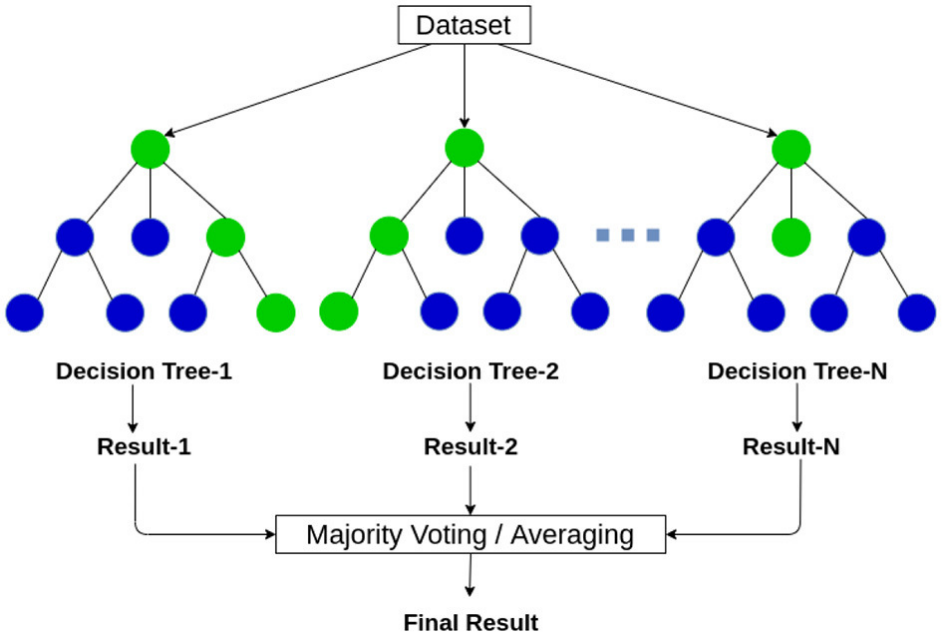
The working of the random forest algorithm (Vidhya, 2023a).

RF is considered a supervised learning classification algorithm which consists of several decision trees, that works based on the majority voting of every single tree when considering a prediction or classification of an entity or an object (Asadi et al., 2021). The functioning method of the mentioned algorithm is demonstrated in Figure 6 The RF classification model utilizes ensemble learning, which is a technique used in machine learning. Ensemble learning acts upon the basis of combining multiple classification models in one classifier to resolve complex categorization and prediction difficulties.

The RF classification model predicts output based on the outcome of several decision trees. It calculates the average of the result of several trees to produce a final prediction. The more count of decision trees more precise the prediction would get. The random forest algorithm eliminates the restrictions in a single decision tree algorithm as it reduces the overfitting of the training dataset, and elevates the precision.

#### 3.4.5 Support vector machine (SVM)

**Figure 7:**
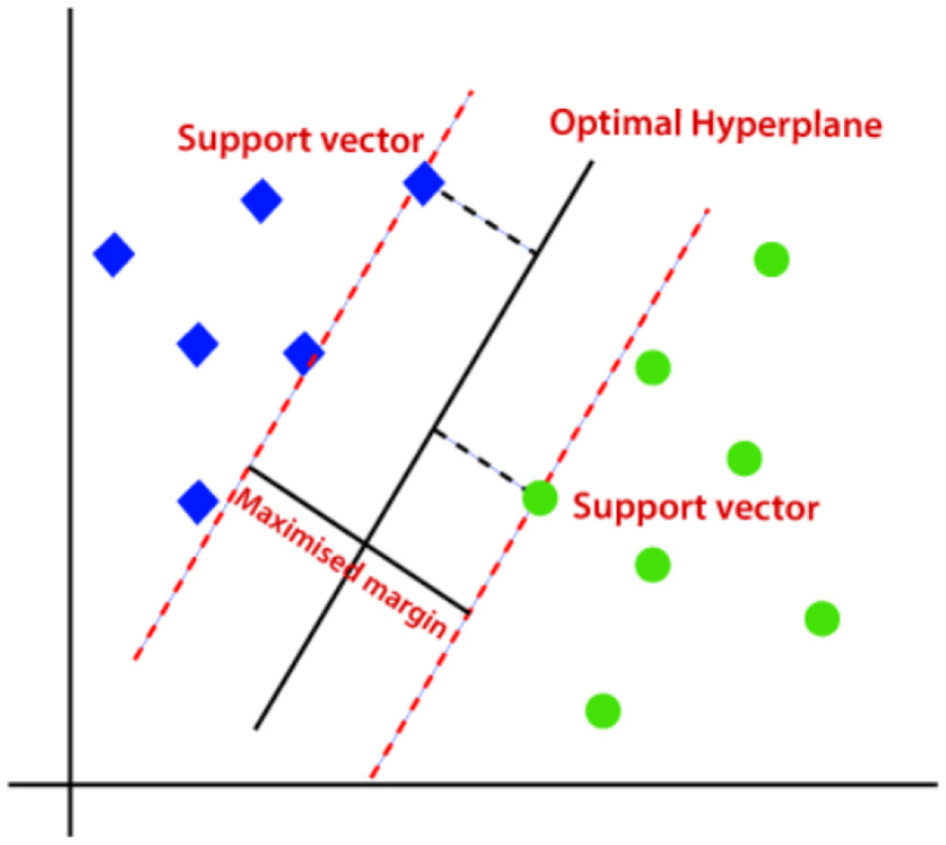
Optimal hyperplane separating two classes (Javatpoint, 2024).

SVM classification model is widely utilized for regression as well as classification problems (Faieq and Mijwil, 2022). Nonetheless, in most cases, SVM is employed for classification difficulties. The aim of the Support Vector Machine classifier is to discover the best boundary stripe or decision line to separate an n-dimension space into multiple categories so it could be feasible to classify new data into its suiting category. This optimal boundary line is called a hyperplane. Figure 7 shows an optimal hyperplane separating two classes.

The optimal decision boundary (Hyperplane) can be constructed or created by utilizing the equation shown in Equation 3 (Rani et al., 2021).


(3) $$\begin{array}{l} w^{T}x+b=0 \end{array}$$

The Support Vector Machine considers the extreme spikes which assist in the formation of a hyperplane. Those extreme samples are called support vectors, hence the classification model is identified as Support Vector Machine. These vectors are referred to as Support vectors since these vectors support the hyperplane.

#### 3.4.6 Multilayer perceptron (MLP)

**Figure 8:**
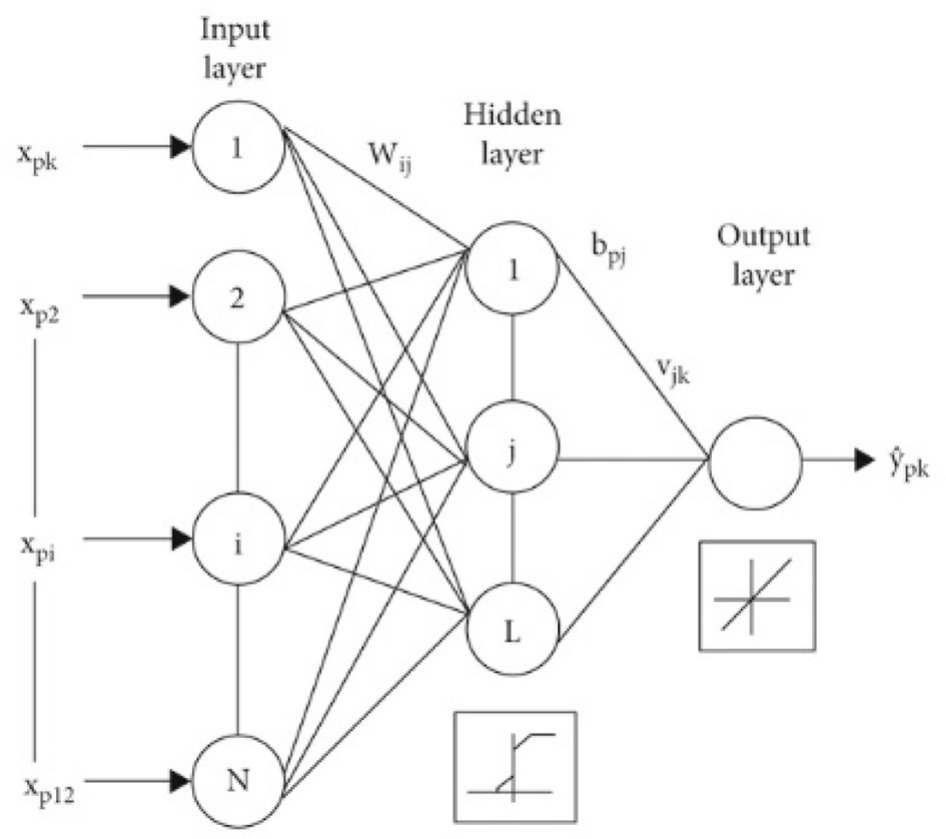
Structure of MLP model (Alnuaim et al., 2022).

This model is a feed-forward artificial neural network model. It contains three main components, which are the input layer in which the input is injected, and the hidden layer which could be more than one layer in which the outcome of the input layer is fed to it (Ahmadian et al., 2022). The last layer is the output layer, in which the classification and prediction are performed, Figure 8 demonstrates the assembly of an MLP classifier. The data is transmitted forward from the input section to the output section, identical to a feed-forward network. Each layer in a multilayer perceptron (MLP) consists of multiple numbers of neurons. These neurons learn patterns in the dataset in the training phase by the backpropagation learning algorithm. The main advantage of the multilayer perceptron (MLP) classification model is that it can solve complex problems that cannot be linearly separated.

#### 3.4.7 Enhanced multilayer perceptron (EMLP)

The EMLP or the enhanced multilayer perceptron model is fundamentally a multilayer perceptron model that has gone through several improvements and modifications in terms of the count of hidden layers, the count of perceptrons in each hidden section, as well as the learning rate.

The first hidden layer on the enhanced multilayer perceptron model consisted of sixty-eight perceptrons which were taking input from the input layer that consisted of sixteen input perceptrons, each perceptron for each feature. The second hidden layer consisted of thirty-four perceptrons which were taking input from the first hidden layer which contained sixty-eight perceptrons. Eventually, the second hidden section was linked to the output section that contained a single perceptron to indicate the presence of heart disease. In the enhanced multilayer perceptron (EMLP) architecture, the learning rate was set to 0.01, which proved to reduce the loss by a noticeable rate and improve the accuracy by 1% from the multilayer perceptron (MLP) classification model.

### 3.5 Evaluation parameters

The experiment outcomes regarding the efficiency of the classification algorithms were evaluated based on the scale of precision, recall, accuracy (Ali et al., 2021), and f1-score achieved by the classification algorithms. If an individual is diagnosed or suffering from heart disease and it was classified by the model it would be considered as a true positive prediction, on the other hand, if it was not detected by the model it would be considered a false negative. If an individual wasn't suffering from heart disease and the classification model predicted or diagnosed that the person is suffering from heart disease, this type of prediction is categorized as false positive otherwise it would be truly negative.

- Accuracy is a performance scale that measures in percentage the accurately predicted data points out of all data points.
-
(4) $$\begin{array}{l} Accuracy=\frac{\left(CorrectPredictions\right)}{\left(TotalPredictions\right)}X100 \end{array}$$
- Precision is the performance scale that measures in percentage count of positive entities predicted actually from the positive class.
-
(5) $$\begin{array}{l} Precision=\frac{\left(TruePositive\right)}{\left(TruePositive+FalsePositives\right)}X100 \end{array}$$
- Recall is the performance scale that measures the positive entities that were correctly predicted as positive to the total number of positive entities.
-
(6) $$\begin{array}{l} Recall=\frac{\left(TruePositive\right)}{\left(TruePositives+FalseNegative\right)}X100 \end{array}$$
- F1-Score (F-Measure) is the performance measure that combines both recall and precision in one metric by computing their harmonic mean.
-
(7) $$\begin{array}{l} F1-Score=2X\frac{\left(Sensitivity*Precision\right)}{\left(Sensitivity+Precision\right)} \end{array}$$

## 4 Results and discussions

Multiple classification algorithms were utilized including the enhanced multilayer perceptron (EMLP) to predict cardiac illness. The CDC's key indicators of the cardiac disease dataset were utilized to execute the experiments. The heart disease diagnosis or prediction was performed using multiple parameters available in the CDC dataset. These parameters or attributes were utilized to classify heart disease with 1 indicating a positive diagnosis of heart disease, and 0 of not suffering from heart disease. The dataset had multiple imbalanced features such as age category, body mass index, mental health, physical health, and sleep time which had to be stabilized to achieve an optimal result in techniques such as multilayer perceptron.

**Figure 9:**
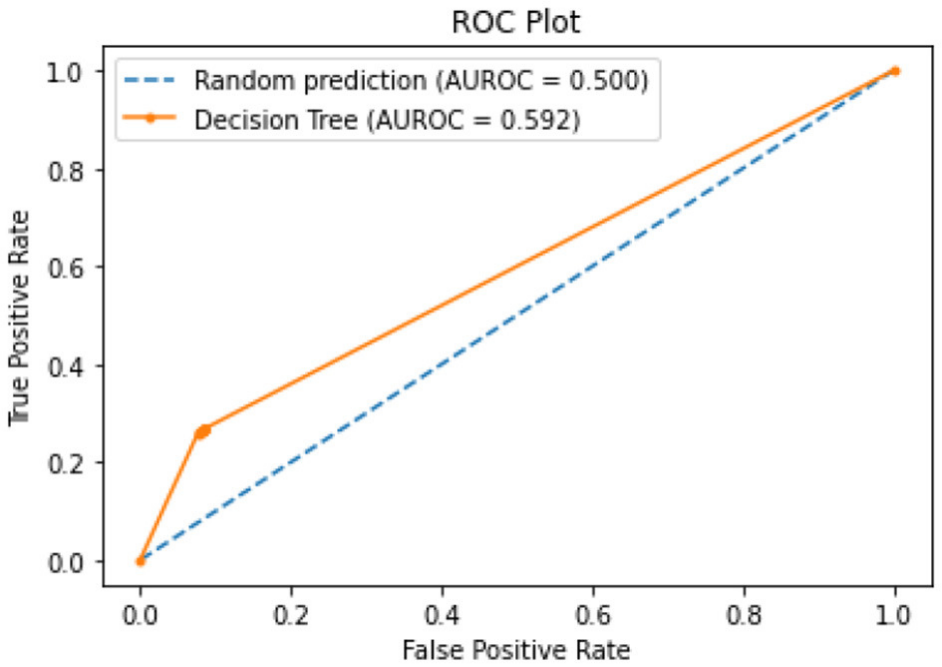
DT model ROC curve.

**Figure 10:**
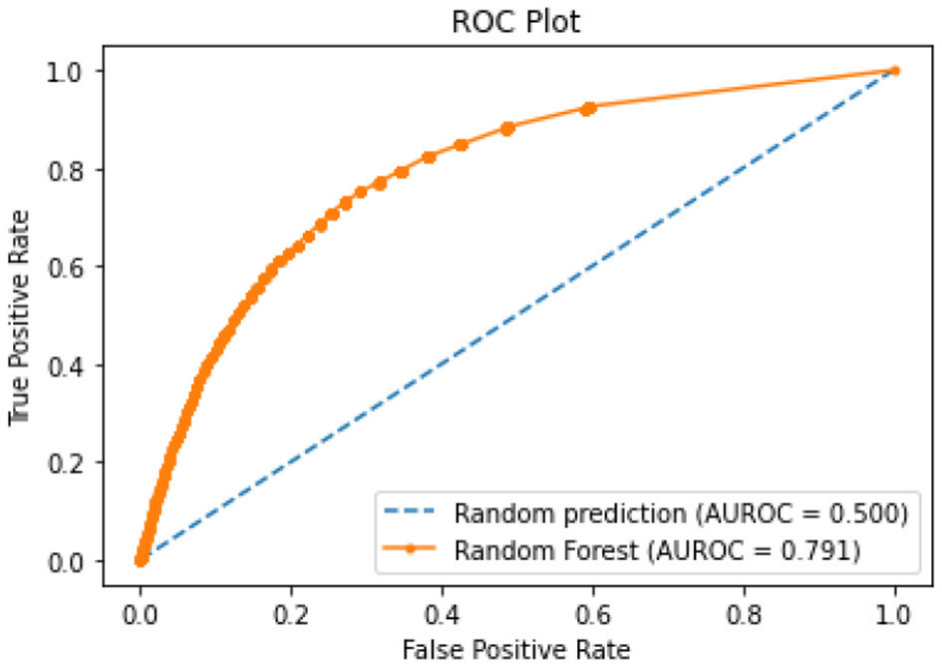
RF model ROC curve.

**Figure 11:**
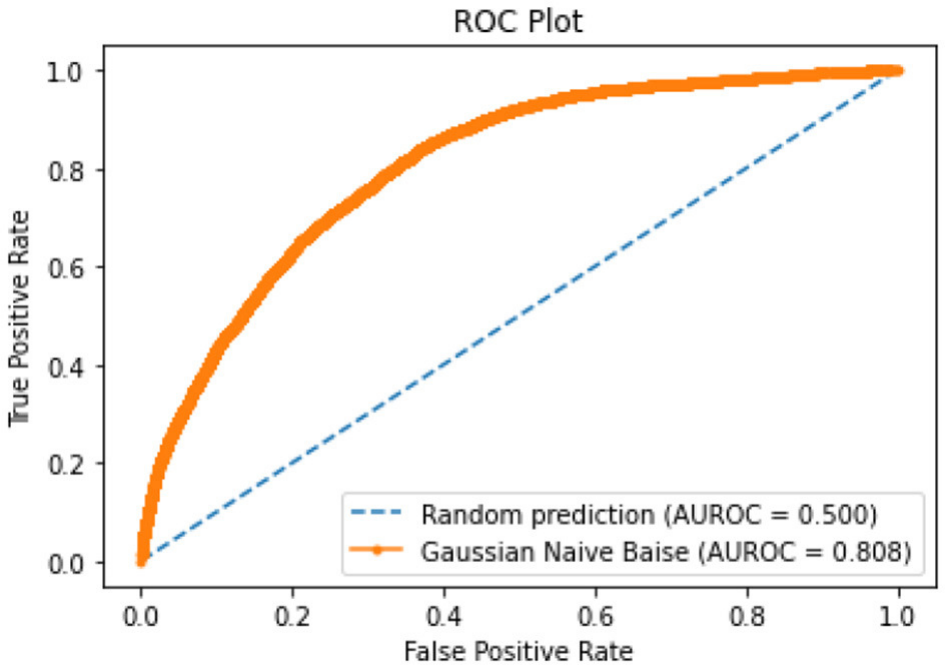
NB model ROC curve.

**Figure 12:**
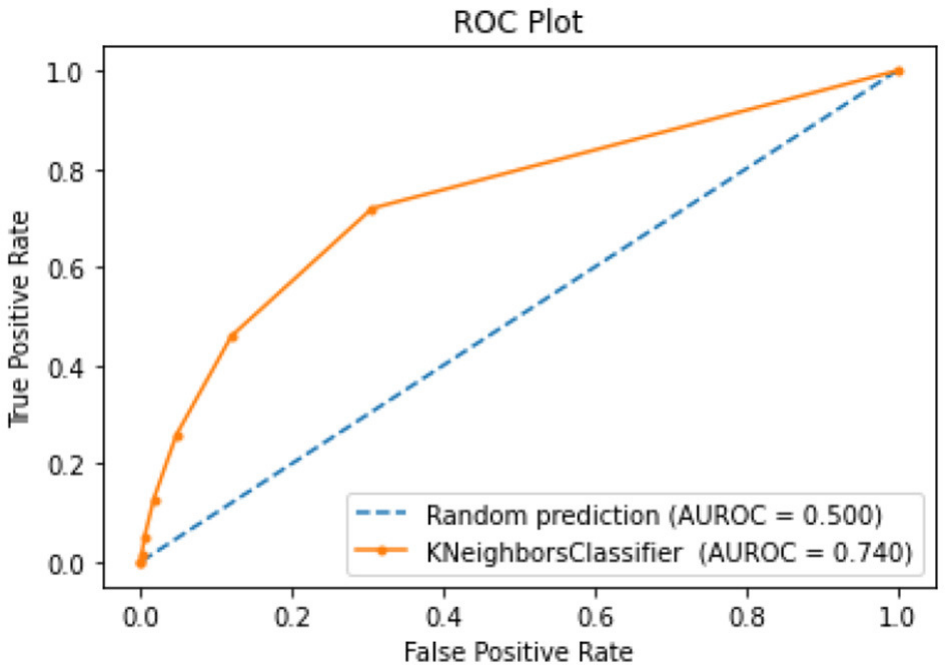
KNN model ROC curve.

**Figure 13:**
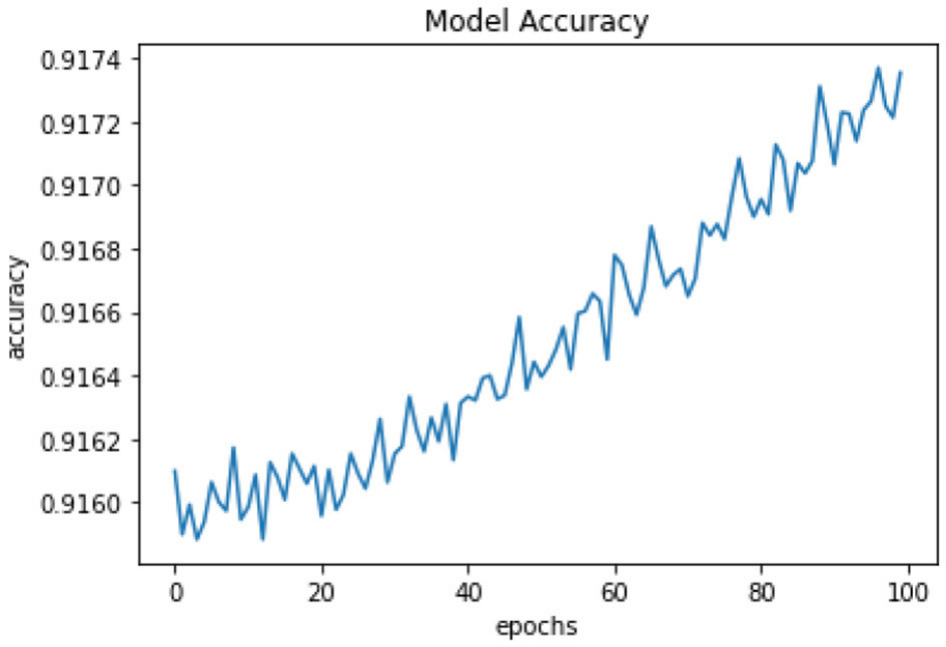
Accuracy graph of the MLP model.

**Figure 14:**
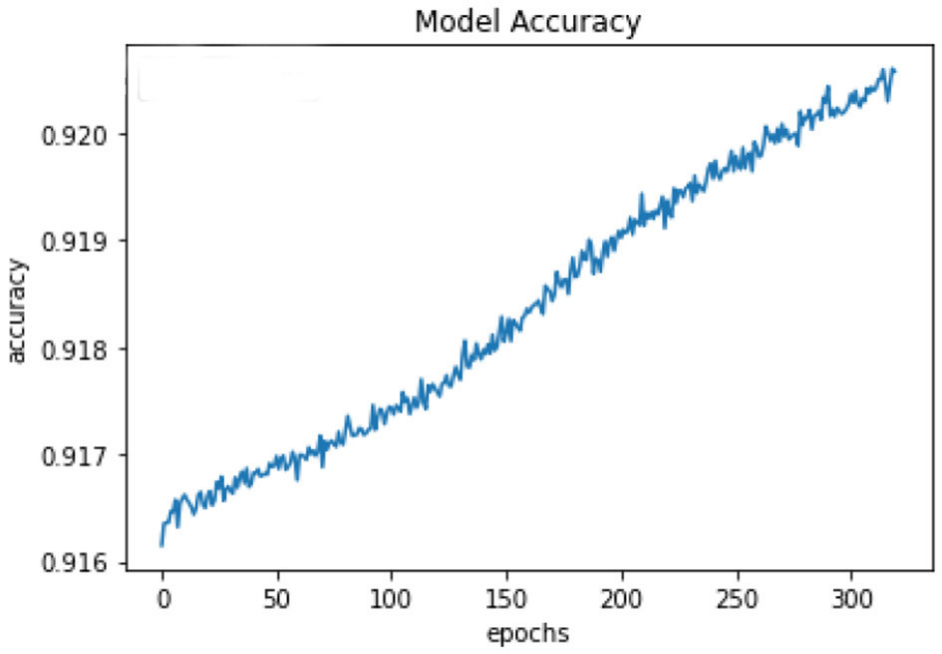
Accuracy graph of the EMLP model.

**Figure 15:**
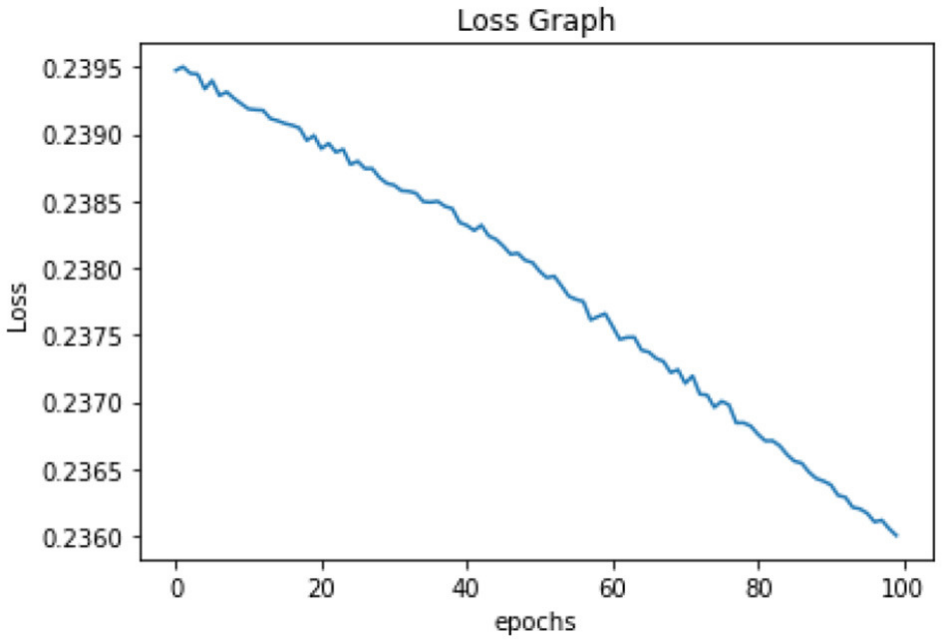
Loss graph of the MLP model.

**Figure 16:**
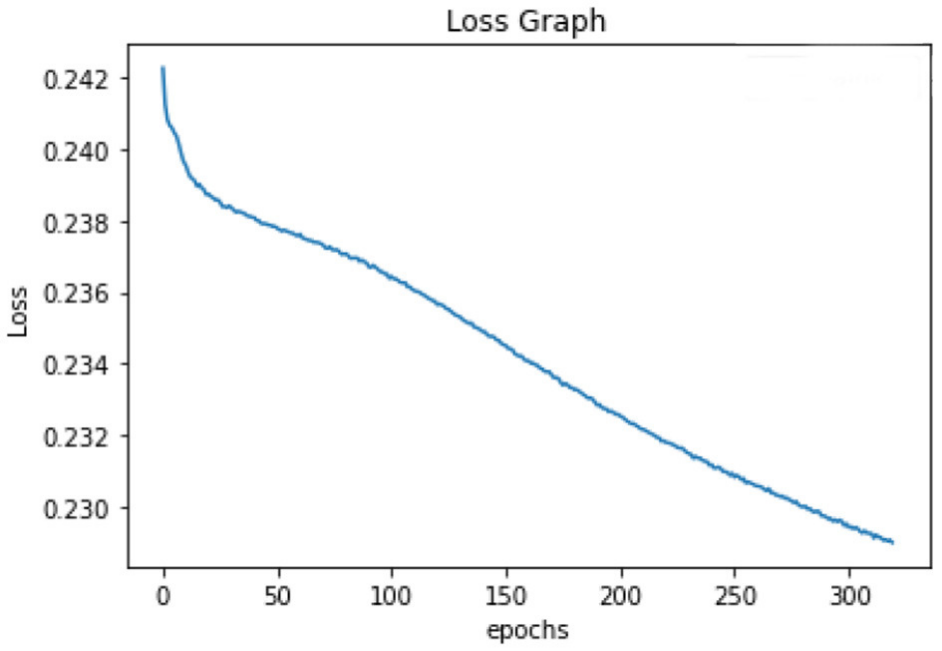
Loss graph of the EMLP model.

All of the suggested classification methods were trained on a separate training dataset created by separating the CDC dataset into a training and testing set. The ROC (Receiver Operating Characteristic Curve) plots in Figures 9–12 demonstrate the training performance of the decision tree classifier, random forest classifier, naïve Bayes classifier, and k-nearest neighbor model. The accuracy of the multilayer perceptron (MLP) and enhanced multilayer perceptron (EMLP) classification models is shown in Figures 13, 14, respectively, and the loss in the multilayer perceptron (MLP) and enhanced multilayer perceptron (EMLP) models is demonstrated in Figures 15, 16, respectively. The evaluation regarding the performance of the classification models that have been employed in this study was assessed by the scale of accuracy, recall, precision, and F1-score.

After processing the dataset to fit our proposed classification models and balancing the imbalanced attributes or features, the outcomes of the experiments are presented in Table 4, which displays the results obtained by the classification models.

**Table 4:** Experimental results.

**Classification models**	**Accuracy (%)**	**Precision (%)**	**Recall (%)**	**F1-score (%)**
Multilayer perceptron (MLP)	91	94	96	95
Decision tree (DT)	86	93	92	92
Random forest (RF)	90	92	98	95
Support vector machine (SVM)	91	91	100	96
K-nearest neighbor (KNN)	91	92	98	95
Naïve Bayes (NB)	84	95	88	91
Enhanced multilayer perceptron (EMLP)	**92**	**95**	**96**	**94**

**Figure 17:**
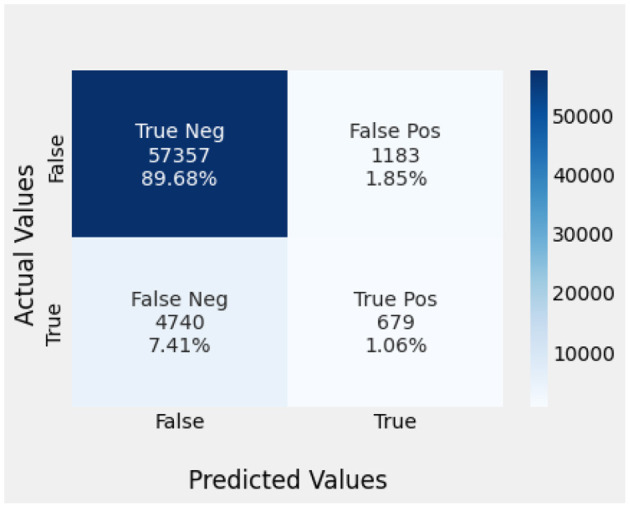
DT model confusion matrix.

**Figure 18:**
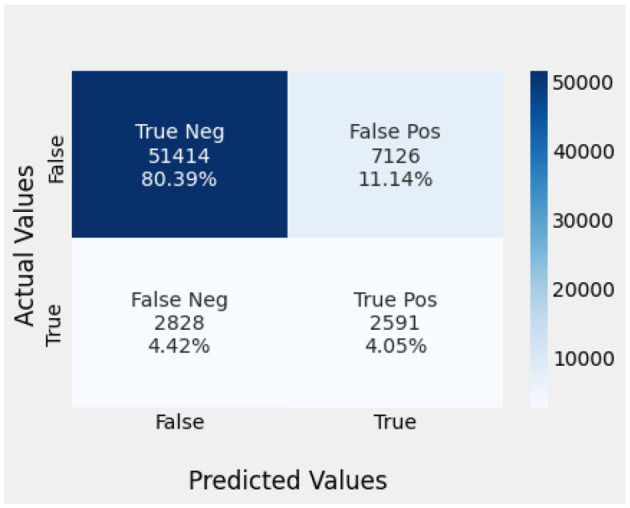
NB model confusion matrix.

**Figure 19:**
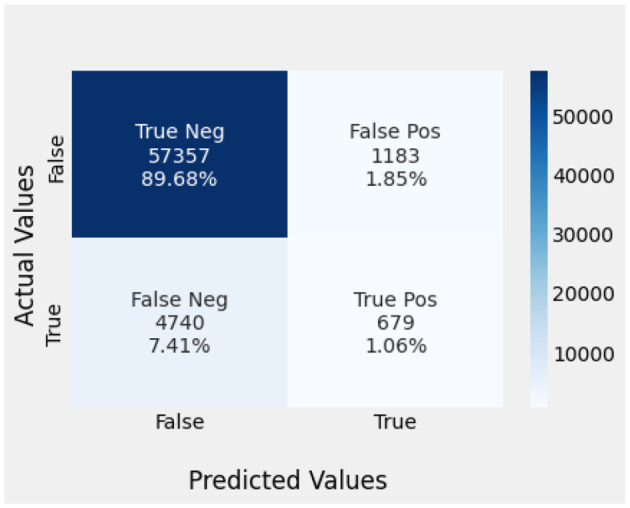
RF model confusion matrix.

**Figure 20:**
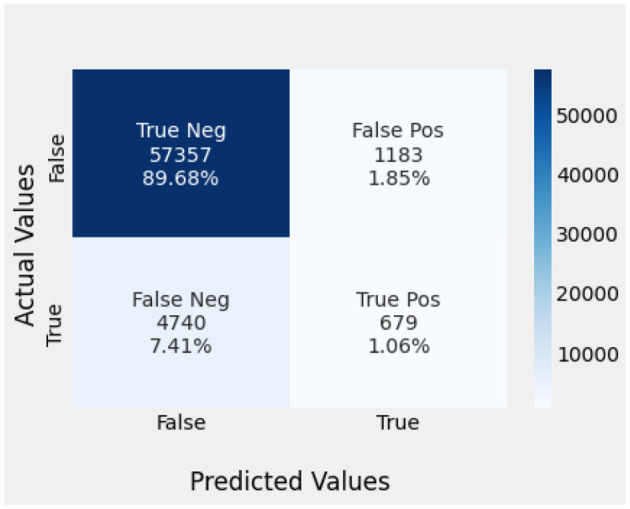
KNN confusion matrix.

**Figure 21:**
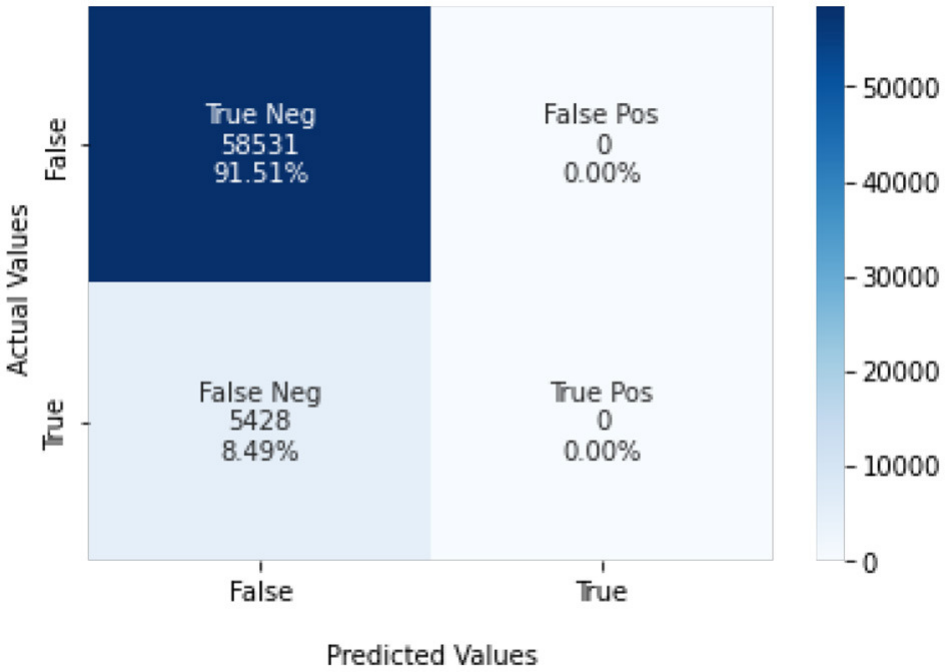
SVM model confusion matrix.

**Figure 22:**
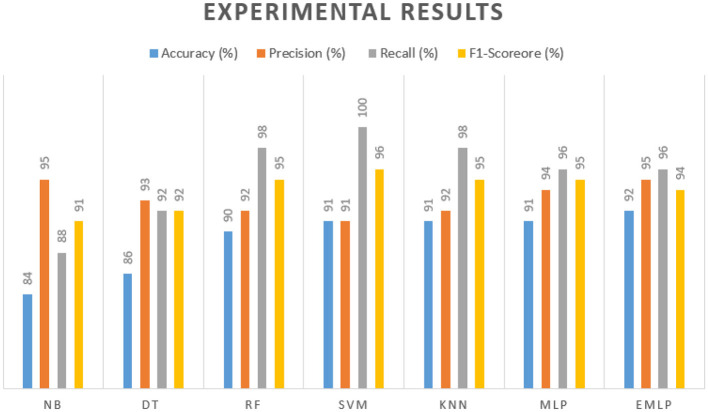
Experimental results of classification models.

Naïve Bayes achieved 84% accuracy, and the decision tree model achieved 86% accuracy, Figures 17, 18 show the result achieved by the decision tree and naive Bayes models respectively in confusion matrices. random forest classifier achieved 90% accuracy, and the k-nearest neighbor model with seven neighbors achieved 91% accuracy in the testing phase. Figures 19, 20 show the result achieved by the random forest and k-nearest neighbor models respectively in the confusion matrices. The support vector machine achieved 91% accuracy in the testing phase. Figure 21 shows the result achieved by the support vector machine model in the confusion matrix. Enhanced multilayer perceptron classifier achieved 92% accuracy. Figure 22 displays the performance of different classification algorithms based on accuracy, precision, recall, and F1-score.

One of the key challenges in deploying AI in healthcare is addressing privacy and security concerns, especially when integrating AI with IoT devices. Shafiq et al. (2024) examined these risks and emphasized the need for robust security frameworks to ensure the safe usage of AI in healthcare environments. The integration of AI in medical diagnostics has immense potential. However, challenges such as privacy concerns, model optimization, and data diversity remain, as highlighted in previous works (Alghamedy et al., 2022; Bilal et al., 2024a; Shafiq et al., 2023).

## 5 Conclusion

Cardiac illness is included among the most dangerous illnesses as it is the main reason for mortality. That is why the timely diagnosis of heart disease will help save lives. To efficiently treat patients before a cardiac attack, it is necessary to properly forecast cardiac illness by employing ML techniques. This study proposes seven ML models to be evaluated on the CDC survey dataset for heart illness prediction. This research shows how by utilizing machine learning algorithms and a few simple clinical indicators available even during the patient's initial visit, the beginning of heart disease can be predicted in an efficient way and timely manner. The collected findings indicate that classifiers based upon enhanced multilayer perceptron are superior in terms of prediction accuracy.

The future work of this study can be performed by employing deep learning techniques, which have proved to perform better in multiple scenarios when compared with machine learning algorithms, but compromising in time complexity as well as space complexity, as it consumes more time while training to achieve much better performance based on prediction/classification accuracy, recall, precision, and f1-score. By Utilizing different technologies like Cloud Computing, big data technologies like Hadoop may be utilized to manage and store massive amounts of data from users all over the world. An Internet of Medical Things (IoMT) system can be developed in future research work, in which an individual can provide symptoms or general attributes on a cloud service, In which the cloud service would pass the person's details through multiple trained machine learning algorithms, that will show the result in an output from. The output will indicate, in the form of a Yes or No answer, whether the subject has a heart condition or not. The outcome will be Yes if the subject is predisposed to having heart disease, and vice versa. In the event of a positive result, a cardiologist should be consulted for a more thorough diagnosis to predict or diagnose heart-related disease in its early stages.

## Data Availability

The original contributions presented in the study are included in the article/supplementary material, further inquiries can be directed to the corresponding author.
